# Reactions of Some New Thienothiophene Derivatives

**DOI:** 10.3390/molecules16065142

**Published:** 2011-06-21

**Authors:** Yahia Nasser Mabkhot, Abdullah Mohammad Al-Majid, Abdullah Saleh Alamary, Ismail Warad, Yamin Sedigi

**Affiliations:** 1 Department of Chemistry, Faculty of Science, King Saud University, P.O. Box 2455, Riyadh 11451, Saudi Arabia; 2 Department of Chemistry, Faculty of Science, Taibah University, P.O. Box 344, Medina, Saudi Arabia

**Keywords:** bis(2**-**bromoethanone), bis**-**thieno[2,3-b]thiophene, bis(oxazole**-**2**-**amine), bis**-**heterocycles, imidazotriazole

## Abstract

Facile and convenient syntheses of bisdimethylthieno[2,3**-**b]thiophen-2,5-diyl bis(oxazole-2-amine), bis(1*H***-**imidazol-2-amine), bis((3a)**-***H***-**indole),[1,2**-**a]pyrimidine), bis(1*H***-**imidazo[1,2**-**b][1,2,4]triazole) and bis(9*H*-benzo[d]imidazo[1,2**-**a]imidazole) derivatives incorporating a thieno[2,3-b]thiophene moiety from the versatile and readily accessible 1,1'(3,4-dimethylthieno[2,3-b]thiophene-2,5-diyl)-bis(2-bromo-ethanone) (**1**) are described

## 1. Introduction

We have been interested for some time in the chemical and biological properties of thienothiophene derivatives [1,2,3]. Thienothiophenes have been developed for different purposes in the pharmaceutical field and have been tested as potential antitumor, antiviral, antibiotic and antiglaucoma drugs or as inhibitors of platelet aggregation [3,4,5,6,7,8]. In addition, thienothiophenes find potential applications in a wide variety of optical and electronic systems [9,10,11]. Recently, some conjugated thienothiophenes, structurally related to several current applications have been reported [12,13,14,15,16,17,18,19]. In continuation of these findings, we report herein the synthesis of some novel bis-heterocycles containing a thieno[2,3-b]thiophene moiety as a base unit and which are of interest as potential biologically active compounds or pharmaceuticals.

## 2. Results and Discussion

**Scheme 1:**
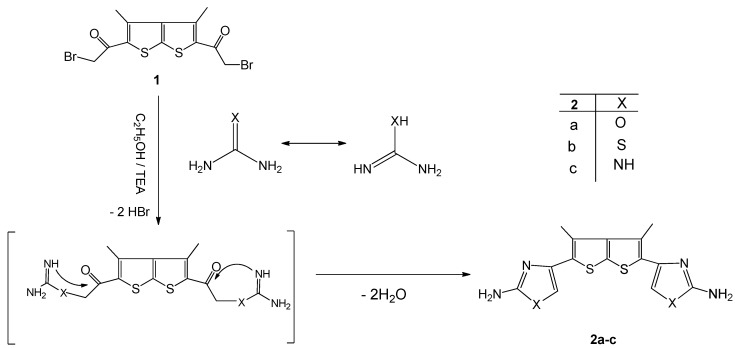
Synthesis of bis-amino heterocycles derivatives **2a-b**.

**Scheme 2:**
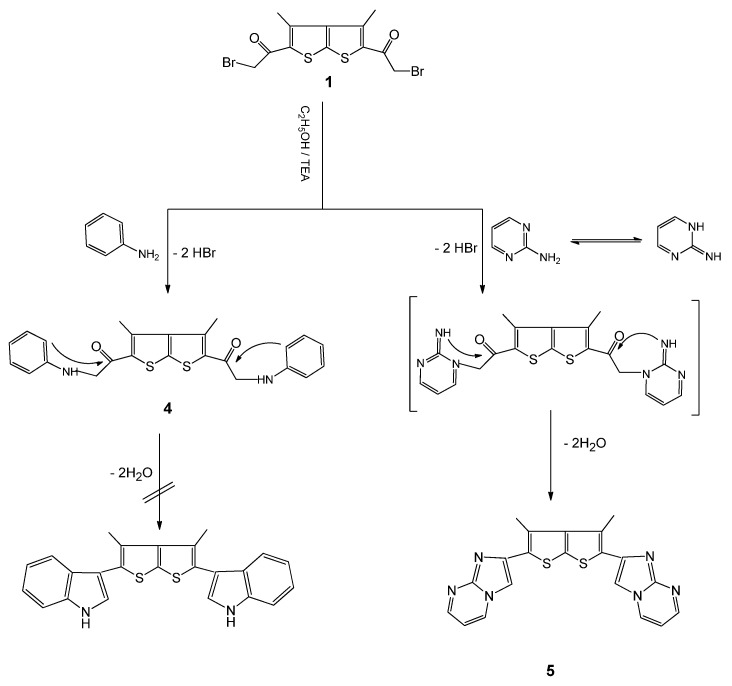
Synthesis of bis- thieno-thiophenes derivatives **4** and **5**.

**Scheme 3:**
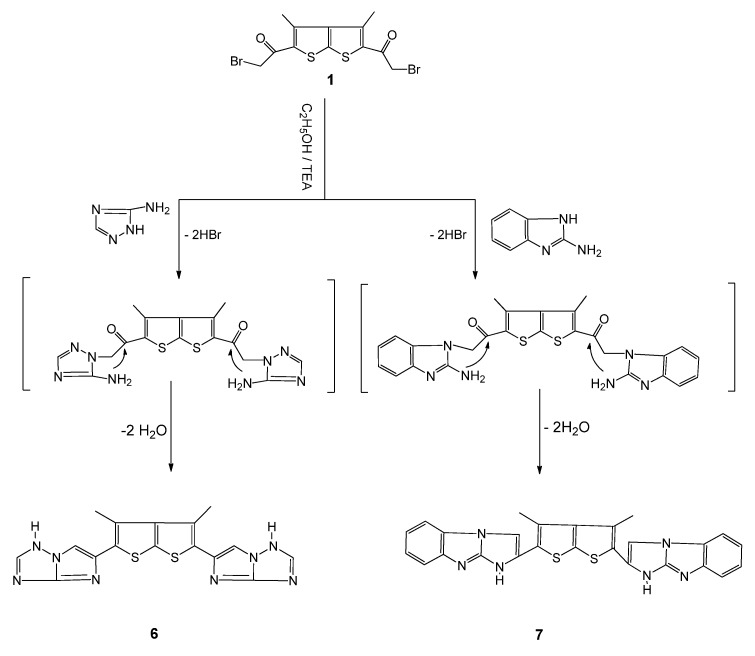
Synthesis of bis- imidazole derivatives **6** and **7**.

The synthetic procedures adopted to obtain the target compounds are outlined in Scheme 1, Scheme 2 and Scheme 3. Treatment of bis-2-bromoacetylthieno[2,3-b]thiophene derivative **1** [3] with urea, thiourea or guanidine in refluxing EtOH/TEA gave the novel bisthieno[2,3-b]thiophene derivatives **2a-c**, respectively (Scheme 1). The structures of the products were deduced from their elemental analysis and spectral data. For example, the ^1^H-NMR spectrum of compound **2a **revealed a singlet at δ 7.19 characteristic of an oxazole CH proton. The IR spectra of **2a-c** showed, in each case, the absence of the carbonyl bands found in **1** and the presence of new bands in the 3422-3385 cm^-1^ region due to NH_2_ and NH groups.

Treatment of compound **1** with aniline or with 2-aminopyrimidine in refluxing EtOH/TEA led to the novel bis-thieno[2,3-b]thiophene derivatives **4** and **5**, respectively (Scheme 2), whose structures were confirmed on the basis of their elemental analyses and spectral data. The ^1^H-NMR spectrum of compound **5**, for example, revealed signals at δ 7.43-7.80, characteristic of imidazole and pyrimidine CH protons. The IR spectrum of **5** lacked a carbonyl absorption band and the ^13^C-NMR spectrum revealed eleven types of carbon atoms (*i.e.*, those of half the bisheterocycle), The IR spectrum of compound **4** showed a carbonyl absorption band at 1690 cm^-1^ [20]. Treatment of compound **1** with 4-amino-1,2,4-triazole in refluxing ethanol afforded 5,5'-(3,4-dimethylthieno[2,3-b]thiophene-2,5-diyl)bis(1H-imidazo[1,2-b][1,2,4]triazole) (**6**, Scheme 3). The **^1^**H-NMR spectrum of compound **6** displayed singlets at δ 2.22 (CH_3_), δ 7.80 (2H, CH, imidazole), 9.8 (s, 2C, CH, triazole) and 12.4 (2H, NH, triazole). The ^13^C-NMR spectrum revealed nine types of carbon. The mass spectrum revealed a molecular ion peak at *m/z* 380, corresponding to C_16_H_20_N_8_S_2_. In a similar manner, when **1** was treated with 2-aminobenzimidazole, the corresponding compound **7** was obtained in high yield.

## 3. Experimental

### 3.1. General

All melting points were measured on a Koffler block melting point apparatus. IR spectra were measured as KBr pellets on a Perkin Elmer FT 1000 spectrophotometer. The NMR spectra were recorded in DMSO-d_6 _ on a Varian Mercury Jeol-(400 MHz) NMR spectrometer. ^1^H-NMR (400 MHz) and ^13^C-NMR were run in (DMSO-d_6_). Chemical shifts were related to that of the residual solvent peak. Mass spectra were recorded on a Shimadzu GCMS-QP 1000 EX mass spectrometer at 70 eV. Elemental analyses were carried out at the Microanalytical Center of King Saud University, Riyadh, Saudi Arabia.

### 3.2. General Procedure for the Reaction of Bis-2-Bromoethanone Derivative ***1*** with Urea, Thiourea and Guanidine: Preparation of Compounds ***2a-c***

Compound **1** (0.410 g, 1 mmol) was treated with urea, thiourea or guanidine (2 mmol) in dry ethanol (20 mL, 99.9%) under reflux for 4-6 h. After addition of TEA (0.5 mL) the corresponding derivatives **2a-c** were formed as solids that were filtered off, washed with ethanol, dried and recrystallized (DMF/EtOH) to afford the desired product in pure form.

*4,4'-(3,4–Dimethylthieno[2,3-b]thiophen-2,5-diyl)bis(oxazole-2-amine)* (**2a**). Dark yellow crystals; yield 77%; mp > 320 °C; IR (KBr) ν_max_ 3417, 3391 (NH_2_) cm^−1^; ^1^H-NMR: δ 2.23 (s, 6H, CH_3_), 7.19 (s, 2H, oxazole), 6.65 (s, 4H, NH_2_ aromatic); ^13^C-NMR: δ 14.8 (2 CH_3_, aliphatic), 128.8, 134.3, 148.1, 148.8 (thienothiophene ArC’s), 136.1, 140.0, 159.3 (ArC’s); MS *m/z* (%): 332 (M^+^, 6), 331 (51), 317 (100), 165 (48), 76 (98). Anal. for C_14_H_12_N_4_O_2_S_2_ (332.40) calcd. C, 50.59; H, 3.64; N, 16.86; S, 19.29. Found: C, 50.48; H, 3.62; N, 16.90; S, 19.20.

*4,4'-(3,4-Dimethylthieno[2,3-b]thiophen-2,5-diyl)bis(thiazol-2-amine)* (**2b**). Bright brown crystals; yield 89%; mp. 295 °C; IR (KBr) ν_max_ 3420, 3391 (NH_2_) cm^−1^; ^1^H-NMR: δ 2.37 (s, 6H, CH_3_), 8.23 (s, 2H, thiazole), 5.88 (s, 4H, NH_2_ aromatic); ^13^C-NMR: δ 15.2 (2 CH_3_, aliphatic), 129.8, 133.8, 147.2, 148.1 (thienothiophene ArC’s), 135.6, 140.2, 167.3 (ArC’s); MS *m/z* (%): 365 (M + 1,15), 364 (M, 39), 331 (51), 207 (48),79 (98). Anal. for C_14_H_12_N_4_S_4_ (364.53) calcd. C, 46.13; H, 3.32; N, 15.37; S, 35.18. Found: C, 46.10; H, 3.34; N, 15.28; S, 35.12.

*4,4'-(3,4-Dimethylthieno[2,3-b]thiophen-2,5-diyl)bis(1H-imidazol-2-amine)* (**2c**). Light brown crystals; yield 95%; mp. 288 °C; IR (KBr) ν_max_ 3422, 3385 (NH_2_), 3220 (NH) cm^−1^; ^1^H-NMR: δ 2.33 (s, 6H, CH3), 7.68 (s, 2H, imidazole), 6.51 (s, 4H, NH_2_ aromatic), 12.28 (s, 2H,NH imidazole). ^13^C-NMR: δ 14.8 (2 CH_3_, aliphatic), 130.5, 134.3, 148.3, 148.6 (thienothiophene ArC’s), 136.3, 141.2, 162.1 (ArC’s); MS *m/z* (%): 331 (M + 1, 28), 330 (M, 100), 298 (21), 168 (43), 98 (63), 79 (38). Anal. for C_14_H_14_N_6_S_2_ (330.43) calcd. C, 50.89; H, 4.27; N, 25.43; S, 19.41. Found: C, 50.78; H, 4.25; N, 25.38; S, 19.36.

### 3.3. General Procedure for the Reaction of Bis-2-Bromoethanone Derivative ***1*** wih Aniline and 2-Aminopyrimidine

Treatment of compound **1** (0.410 g, 1 mmol) with aniline or 2-aminopyrimidine (2 mmol) in dry ethanol (20 mL 99.9%) at reflux for 5-8 h afforded the corresponding derivatives **4** and **5**, respectively. The solid products formed were filtered off, washed with ethanol, dried and recrystallized (DMF/EtOH).

*2,2'-(3,4-Dimethylthieno[2,3-b]thiophen-2,5-diyl)bis((3a)H-indole))* (**4**). Yellow crystals; yield 95%; mp > 320 °C; IR (KBr) ν_max_ 3390 (NH), 1690 (C=O) cm^−1^; ^1^H-NMR: δ 2.83 (s, 6H, CH_3_), 7.77-7.83 (s, 10H, ArH’s); 6.33 (2H, NH); ^13^C-NMR: δ 15.1 (2 CH_3_, aliphatic), 131.3, 132.3, 144.6, 148.3 (thieno-thiophene ArC’s), 34.9, 105.3, 121.2, 122.2, 124.6, 127.2, 166.4 (ArC’s); MS *m/z* (%): 399 (M + 1, 41), 398 (M, 89), 397 (84), 383 (24),165 (54). Anal. for C_24_H_18_N_2_S_2_ (398.54) calcd. C, 72.33; H, 4.55; N, 7.03; S, 16.09. Found: C, 72.22; H, 4.49; N, 7.13; S, 16.03.

*2,2'-(3,4-Dimethylthieno[2,3-b]thiophen-2,5-diyl)bis(imidazo[1,2-a]pyrimidine)* (**5**). Brown crystals; yield 78%; mp > 320 °C; IR (KBr) ν_max_ 1600 (C=N) cm^−1^; ^1^H-NMR: δ 2.97 (s, 6H, CH_3_), 7.78 (s, 2H, CH, imidazole), 7.43, 7.45, 7.80 (s, 6H, CH, pyrimidine); ^13^C-NMR: δ 14.3 (2 CH_3_, aliphatic), 130.2, 133.4, 145.7, 148.4 (thienothiophene ArC’s), 103.9, 111.4, 122.2, 127.2, 159.1, 163.2 (ArC’s); MS *m/z* (%): 403 (M + 1,46), 402 (M, 100), 387 (29), 284 (12). Anal. for C_24_H_18_N_2_S_2_ (402.50) calcd. C, 59.68; H, 3.5; N, 20.88; S, 15.93. Found: C, 59.56; H, 3.48; N, 20.78; S, 15.99.

*5,5'-(3,4-Dimethylthieno[2,3-b]thiophen-2,5-diyl)bis(1H-imidazo[1,2-b][1,2,4]triazole* (**6**). Compound **1** (0.410 g, 1 mmol), was added to 4-amino-1,2,4-triazole (0.168 g, 2 mmol) in dry ethanol (20 mL, 99.9%) at reflux for 4 h. After adding TEA (0.5 mL), two minutes of heating followed. The solid product formed was filtered off, washed with ethanol, dried and recrystallized (DMF/EtOH). Red crystals; yield 81%; mp. 228-230 °C; IR (KBr) ν_max_ 3385 (NH) 1560 (C=N) cm^−1^; ^1^H-NMR: δ 2.22 (s, 6H, 2 CH_3_), 8.52 (s, 2H, CH, imidazole) 9.8 (s, 2H, CH, triazole), 12.4 (2H, NH, triazole); ^13^C-NMR: δ 15.4 (2 CH_3_, aliphatic), 137.8, 140.9, 144.4, 148.1 (thienothiophene ArC’s), 120.1, 124.1, 156.5, 163.0 (ArC’s) MS *m/z* (%): 381 (M + 1, 13), 380 (M, 100), 378 (22), 365 (36), 98 (14). Anal. for C_16_H_20_N_8_S_2_ (380.06) calcd. C, 50.51; H, 3.18; N, 29.45; S, 16.86. Found: C, 50.46; H, 3.17; N, 29.22; S, 16.77.

*2,2'-(3,4-Dimethylthieno[2,3-b]thiophen-2,5-diyl)bis(9H-benzo[d]imidazo[1,2-a]imidazole)* (**7**). Compound **1** (0.410 g, 1 mmol), was added to 2-aminobenzimidazole (0.266 g, 2 mmol) in dry ethanol (20 mL, 99.9%) at reflux for 6 h. After adding TEA (0.5 mL), two minutes of heating followed. The solid product so formed was filtered off, washed with ethanol, dried and recrystallized from (DMF/EtOH). Yellow crystals; yield 78%; mp > 320 °C; IR (KBr) ν_max_ 3414 (NH), 1544 (-C=N) cm^−1^; ^1^H-NMR: δ 2.30 (s, 6H, 2 CH_3_), 8.86 (2H, imidazole C-H), 12.8 (2H, NH, imidazole), 7.31, 7.33, 7.35 (4H, CH, benzimidazole); ^13^C-NMR: δ 15.88 (2CH_3_, aliphatic), 137.8, 140.9, 144.4, 148.1 (thienothiophene ArC’s) 107.1, 112.5, 124.1, 124.5, 124.9, 125.0, 157.2 (ArC’s); MS *m/z* (%): 479 (M + 1, 35), 478 (M, 10), 476 (54), 318 (21), 96 (86). Anal. for C_26_H_18_N_6_S_2_ (478.59) calcd. C, 65.25; H, 3.79; N, 17.56; S, 13.40. Found: C, 65.22; H, 3.67; N, 17.62; S, 13.33. 

## 4. Conclusions

Syntheses and identification of some bis-heterocycles **2a-c** and **4-7** containing thieno[2,3-b]thiophene moieties *via* the versatile, hitherto unreported reagent 2-bromo-1-[5-(2-bromoacetyl)-3,4-dimethyl-thieno[2,3-b]thiophen-2-yl]-ethanone (**1**) were reported.
